# Retrieval with gene queries

**DOI:** 10.1186/1471-2105-7-220

**Published:** 2006-04-21

**Authors:** Aditya K Sehgal, Padmini Srinivasan

**Affiliations:** 1 Department of Computer Science, The University of Iowa, Iowa City, IA 52246, USA; 2 School of Library and Information Science, The University of Iowa, Iowa City, IA 52246, USA

## Abstract

**Background:**

Accuracy of document retrieval from MEDLINE for gene queries is crucially important for many applications in bioinformatics. We explore five information retrieval-based methods to rank documents retrieved by PubMed gene queries for the human genome. The aim is to rank relevant documents higher in the retrieved list. We address the special challenges faced due to ambiguity in gene nomenclature: gene terms that refer to multiple genes, gene terms that are also English words, and gene terms that have other biological meanings.

**Results:**

Our two baseline ranking strategies are quite similar in performance. Two of our three LocusLink-based strategies offer significant improvements. These methods work very well even when there is ambiguity in the gene terms. Our best ranking strategy offers significant improvements on three different kinds of ambiguities over our two baseline strategies (improvements range from 15.9% to 17.7% and 11.7% to 13.3% depending on the baseline). For most genes the best ranking query is one that is built from the LocusLink (now Entrez Gene) summary and product information along with the gene names and aliases. For others, the gene names and aliases suffice. We also present an approach that successfully predicts, for a given gene, which of these two ranking queries is more appropriate.

**Conclusion:**

We explore the effect of different post-retrieval strategies on the ranking of documents returned by PubMed for human gene queries. We have successfully applied some of these strategies to improve the ranking of relevant documents in the retrieved sets. This holds true even when various kinds of ambiguity are encountered. We feel that it would be very useful to apply strategies like ours on PubMed search results as these are not ordered by relevance in any way. This is especially so for queries that retrieve a large number of documents.

## Background

This research focuses on the problem of retrieval from MEDLINE for gene queries. The ability to retrieve the correct set of documents about genes is at the foundation of a fast growing variety of text-based solutions in bioinformatics. Whether the end goal is to automatically identify gene disease relationships [1] or to extract relevant information about drugs and genes related to a specific disease such as cancer [2], it is important to identify the associated document set accurately. Effective retrieval for gene queries is particularly relevant to the expanding body of research on using MEDLINE to analyze gene clusters generated by DNA microarray and oligonucleotide arrays experiments [3-7]. A single array experiment typically involves thousands of genes, often on a genome-wide scale. This makes the analysis of array expression data quite challenging. Several researchers have proposed techniques using MEDLINE data for each gene. For example Chaussabel and Sher [7] complement gene expression cluster analysis by clustering genes according to their literature profiles. Kankar et al. [5] statistically evaluate MeSH terms from the gene literature to identify a gene cluster's topical characteristics at different levels of importance. Wren and Garner [6] exploit literature co-occurences to evaluate the cohesiveness of gene clusters. Naturally such methods for analyzing several hundred genes at a time crucially depend upon the accuracy of their underlying document retrieval functions.

A significant aspect that makes gene query retrieval challenging, and hence interesting, is the ambiguity associated with gene names [8]. There is a sizable and growing body of research on this gene name ambiguity phenomenon [9-13], with particular emphasis on designing and testing disambiguation strategies. Weeber et al. [11] studied the various kinds of ambiguities, such as synonymy and homonymy, in LocusLink (LL) gene names. They also use the Schwartz and Hearst expansion algorithm to automatically create a gene disambiguation test collection. Tuason et al. [12] studied ambiguity in gene names for four organisms (Mouse, Drosophila, Worms and Yeast). They identify ambiguity across all organisms, within each organism and with general English words.

Various disambiguation approaches can be found in the literature. Liu et al. [14] used a two-phase unsupervised approach to automatically train and build word sense classifiers for ambiguous biomedical terms. Podowski et al. [15] used a supervised approach to assign to each gene name in a MEDLINE abstract its corresponding LocusLink ID. They created models for each LocusLink ID trained using MEDLINE citations in LocusLink and SWISSPROT records. Koike and Takagi [16] used heuristically built dictionaries for gene names and for gene family names while Seki and Mostafa [17] explored probabilistic approaches. Most recently Schijvenaars et al. [18] used a method involving both a thesaurus and reference descriptions for the different meanings of genes with the latter built from representative documents or from OMIM. Their thesaurus of gene symbols, names etc. was built from five public databases such as OMIM and LocusLink.

As indicated before, our approach is to view the problem from a *retrieval *perspective. Initiatives such as the KDD 2002 challenge cup [19], BioCreAtIvE challenge [20] and TREC Genomics [21] offer related research. However, our research is distinct both in its goals and in the experimental design. In KDD 2002 although one task was to rank and retrieve papers in order of probability of the need for curation, the experimental conditions are significantly different from ours. For example, the collection had less than 1100 "cleaned" full text papers from the FlyBase domain. In sub-task 2.3 for the BioCreAtIvE 2004 workshop, participants were asked to "provide for, ten proteins, the articles which are relevant for annotation," along with information pertaining to GO annotation [22]. Here again the collection was limited to 212 full text articles from the Journal of Biological Chemistry. Moreover, as stated by the organizers, the results of the sub-task were not evaluated due to reasons such as "the limited number of participants".

In the 2003 TREC Genomics Track, one task was retrieval from a collection of 525,938 MEDLINE records for 50 gene topics [23]. The 2004 TREC Genomics track also had a retrieval task again with 50 queries but this time representing a broader variety of bioinformatics queries [24] as for example queries exploring the relationship between a gene and a disease. Our effort is different from these two TREC efforts. Although we use a dataset of 4.6 million MEDLINE records, built chiefly from the TREC 2004 dataset, we focus on a much larger (close to 9,400 queries) and different query set (focussed on gene queries). We use a similar, but not identical strategy, as that in TREC 2003, to identify gold standard relevant documents. Our method identifies more than double the number of relevant documents. In addition to differences in experimental design, our retrieval goal relates more directly to the needs of bioscientists using MEDLINE based evidence for the analysis of array based expression data involving thousands of genes.

In contrast to the extensive research on gene string ambiguity recognition and resolution, there are few studies where the central emphasis is on assessing the effectiveness of MEDLINE document *retrieval *with gene queries. Disambiguation focuses on individual occurrences of gene strings in specific documents. However, although a disambiguation strategy may correctly decide that a given ambiguous string represents a gene of interest, the document may still be non relevant. This could happen for example if the gene was mentioned only in a peripheral context. Retrieval on the other hand is, by definition, concerned with relevance. In the long run it may be beneficial to combine the strengths of both retrieval research and disambiguation research when working with genes. Our focus in this paper is on retrieval. For each gene in our sample we begin with the set of documents retrieved via PubMed and aim at improving this set using ranking methods from information retrieval research.

PubMed, the public interface to MEDLINE, offers a sophisticated range of search functions designed within the Boolean framework. However, the main option for sorting a retrieved set of documents is chronological. In other words, a PubMed query divides the MEDLINE collection into 2 sets: one that satisfies the query and one that does not. The former is then shown to the user in chronological order. There are no 'shades of grey' in PubMed retrieved sets. Ranking documents by their relevance potential can be of significant benefit, especially when large sets of documents are retrieved – the case with many gene queries. Thus our objective is to explore strategies for effectively *ranking *documents retrieved by PubMed. We begin with a baseline ranking strategy that uses only the terms in the original gene query submitted to PubMed. We then explore a variety of other ranking strategies assuming different levels of domain knowledge about the genes. We also study the effect of ambiguity on performance.

In recent research, Chen et al. [13] conducted the most extensive study to date, on gene names. Exploring 21 species, they studied the distribution of different ambiguities including the ones studied in this research. They found, for example, that although only 0.57% of their gene set consisted of genes with English meanings (in the context of a mouse dataset), these retrieved an additional 233% gene document 'instances' of which the majority were incorrect. We offer logically complementary research albeit one that focuses on the genes of a single genome. We present a systematic study on retrieval effectiveness, for human genes, with all of their inherent nomenclature ambiguities. Specifically we present three main experiments altogether involving 9,390 genes (human genes with known function identified from LocusLink (LL)). Each experiment explores the effectiveness of one or more ranking queries applied to document sets retrieved for gene queries from MEDLINE. Our goal is to rank relevant documents higher than the non relevant ones in the retrieved set.

## Results and discussion

For each gene we retrieve documents from MEDLINE by searching the disjunction of its aliases taken from the OFFICIAL_GENE_NAME, OFFICIAL_SYMBOL and ALIAS_SYMBOL fields of LL. Retrieved documents are restricted to a subset of MEDLINE (close to 4.6 million records) consisting chiefly of the 2004 TREC Genomics dataset [24]. 44% of the gene queries retrieve 100 or more documents while almost 25% retrieve 500 or more documents. Relevant documents (our gold standard) are extracted from the PMID' and 'GRIF' fields in each gene's LL record. In TREC 2003, gold standard documents were identified for the 50 gene topics using only the GRIF field of LL. This pool of relevance information has been noted to be incomplete [23]. For our collection of 9,390 queries, our strategy extracting from both PMID and GRIF fields identified more than twice the number of gold standard judgments (47,639) as compared to using the GRIF field alone (21,517). We observe that 76% of our topics have five or less relevant documents identified through LL. This suggests that ensuring accuracy in retrieval for gene queries is a challenge and users are likely to benefit from retrieved sets ranked by relevance potential. For a given gene we compute a set of term vectors using a basic tf*idf strategy for term weighting. Term vectors are computed for each retrieved MEDLINE record and for each ranking query. Cosine similarity scores in [0,1] are calculated for each ranking query vector – retrieved document vector pair. Given a ranking query retrieved documents are ranked by cosine similarity to the query. The ranked sets are limited to the top ranked 10,000 documents. We believe it is unlikely that a user would want a larger retrieved set. We measure the quality of ranking using average precision (AP) [24]. AP is the average of the precision scores calculated at the position of each relevant document in a ranked document list. E.g., given a ranked list where the 3 gold standard documents are at rank 2, 5 and 7, AP is the average of precision scores (0.5, 0.4, 0.43) which equals 0.44. Since AP is sensitive to the rank of each relevant document we also compute (normalized) precision of the top 5 ranked documents (NTop5P = Top5P/max_Top5P). Top5P is the number of relevant documents in the top 5 ranks divided by 5. The normalization factor is included as some queries have less than 5 relevant documents. E.g., if a gene has only 3 gold standard documents then the max_Top5P is 0.6. If for such a query, all 3 relevant documents are within the top 5 positions, NTop5P = 0.6/0.6 = 1. If instead only 2 are in the top 5, then NTop5P = 0.4/0.6 = 0.63. For queries with at least 5 relevant documents, NTop5P is the same as Top5P.

Scores are averaged across topics to yield mean AP (MAP) and mean NTop5P. AP is our primary measure. We compare five document ranking strategies. B1 and B2 are baseline strategies. S, P and SP are built from LL.

1. **Baseline 1 (B1)**: This ranking query is the same as the PubMed query (gene name and aliases) without the disjunction operator.

2. **Baseline 2 (B2)**: We add to the B1 ranking query the terms 'gene', 'genetics', 'genome' and 'oncogene'. Here we hope to steer the ranking in favor of documents in the overall genetics domain. This query is motivated in part by the notion of a "query zone" [25].

3. **Summary (S)**: We add to B1 the SUMMARY field of the gene's LL record. This field, when available, describes for example, the gene's function, its structure and associated phenotype information. It is generated using data from various sources [26].

4. **Product (P)**: We add to the B1 query the PRODUCT, PREFERRED_PRODUCT, ALIAS_PROT fields in LL.

5. **Summary+Product (SP)**: Both LL summary and product information are added to the B1 ranking query.

Unfortunately not all 9,390 genes in our pool have both the summary and product fields in LL. A subset of 4,647 genes have both summary and product (used in expt. 1); a different subset of 4,195 has no summary (used in expt. 2), while the full set is used in expt. 3.

### Ranking results (Expt. 1)

**Figure 1:**
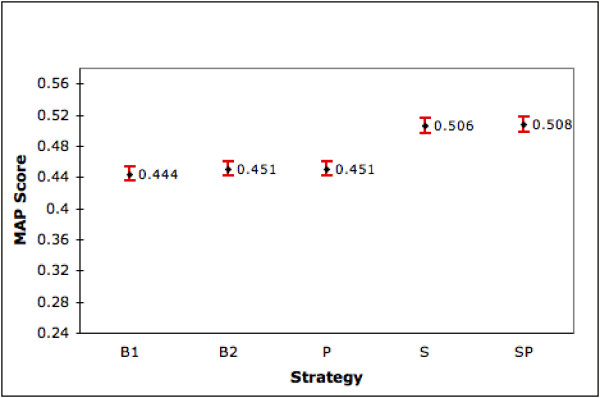
**Performance of Ranking Strategies (MAP)**. The graph shows the mean AP scores (with 95% confidence interval) for the different strategies on the set of 4,647 genes for which summary and product is available in LL.

**Figure 2:**
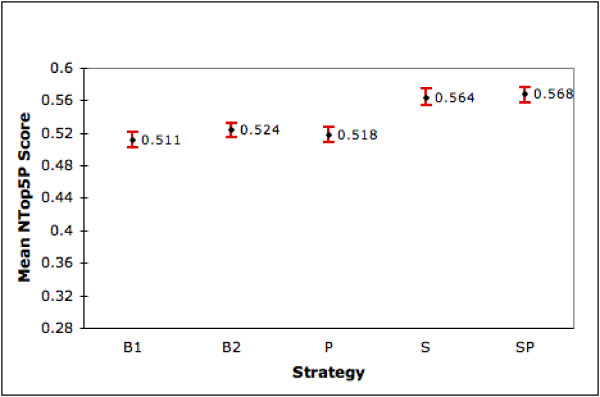
**Performance of Ranking Strategies (NTop5P)**. The graph shows the mean NTop5P scores (with 95% confidence interval) for the different strategies on the set of 4,647 genes for which summary and product is available in LL.

We first compare our five ranking strategies using the 4,647 genes that have both summary and product fields. Figure 1 shows the *MAP *scores for each strategy along with the 95% confidence intervals. We find that the generic query (B2) is not that different from B1 in performance. But S and SP give significant gains in the range of 14% compared to B1 and 12% compared to B2. Additionally, the 95% confidence intervals for S and SP do not overlap with those for B1 and B2 indicating that the differences are statistically significant at the 0.05 significance level. P is weak even when compared with B1 although it does not hurt performance when added to S (S and SP show almost identical results). Figure 2 shows the corresponding mean NTop5P scores for each strategy. Again we see that S and SP are significantly different from B1 and B2 at the 0.05 significance level. Improvements are around 10.4% and 8% compared to B1 and B2 respectively.

**Figure 3:**
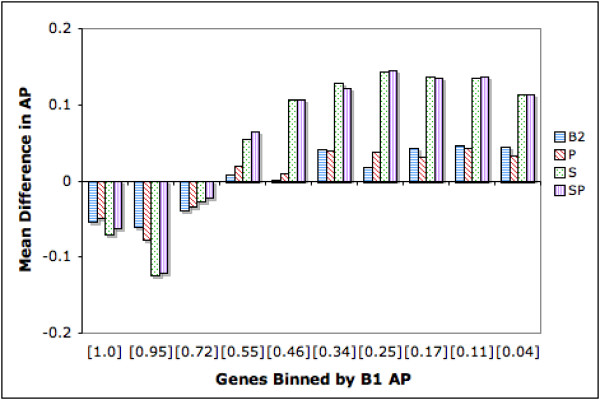
**Difference in Average Precision: Genes Binned by B1 AP**. The genes are distributed into 10 bins defined by B1 AP score. Each bin has 450 genes except for the right most bin, which has 617 genes. Average B1 scores for the genes in the bins are shown in square brackets along the X axis. The Y axis depicts the mean difference in AP between a given strategy and B1. Thus for example, for the bin closest to the origin, which has average B1 score of 1.0, B2 degrades performances. On average it brings down AP by 0.06. Bars below the X axis indicate negative effects of ranking and bars above indicate positive effects. The height of the bars indicate the extent of the improvement/drop in performance.

For more detailed analysis, figure 3 displays difference graphs for AP scores where differences are calculated against B1. The genes are distributed into 10 bins defined by B1 AP score. We see that as B1 performance drops approximately below 0.7, the other ranking strategies generally become increasingly beneficial. In fact the best strategy, SP yields increases in AP from 0.06 to 0.14 from the 4th bin onwards to the right. Assessed against the baseline averages, this bin range showing significant improvements, spans 3,297/4,647 (71%) of the genes. Again we see that P is not effective.

**Figure 4:**
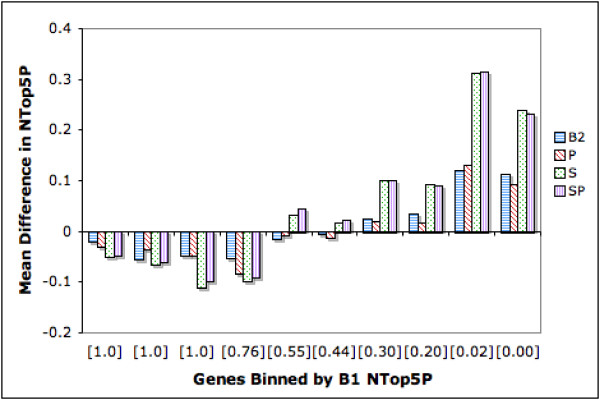
**Difference in NTop5 Precision: Genes Binned by B1 NTop5P. **The genes are binned by B1 NTop5P Score. Each bin has 450 genes except for the right most bin, which has 617 genes. The average B1 NTop5P score for each bin is shown in square brackets along the X axis. The Y axis indicates the mean difference in NTop5P between a given strategy and B1. Bars belowthe X axis indicate a drop in performance whereas bars above the X axis indicate an improvement in performance. The extent of improvement/drop is indicated by the height of the bars.

Figure 4 shows difference plots for NTop5P scores. Binning is again done by the corresponding B1 score. We see that as the baseline performance drops below 0.7, other ranking strategies show positive effect. SP is the best with average increases in the range of 0.02 to 0.3 from bin 5 onwards. Here too, P does not perform well.

In general, our best strategies, SP and S, do significantly better than B2. For SP, the percentage improvements start at 11.6% for AP (from bin 4) and 7.9% for NTop5P (from bin 5). Thus our gene specific strategies perform better than the generic ranking strategy. Not surprisingly, we observe that it is difficult to make improvements when B1 performance is already quite reasonable. The question we now face is, given a gene query, can we predict whether its B1 performance is going to be sufficient? In other words, can we identify genes for which our SP ranking will generate improvements? We return to this question later in the paper.

### Results for ambiguous genes

A significant emphasis in recent research is on exploring and understanding the extent and variety of ambiguity in gene terms. Thus we examine the merits of our ranking strategies within particular genes that are referenced by ambiguous terms. We explore three varieties of ambiguity. These are not mutually exclusive, since a given gene search term may be ambiguous in more than one way. We remind the reader of the distinction between a gene and its component gene terms (search terms). Ambiguities are determined at the search term level. A gene is considered ambiguous if at least one of its search terms is ambiguous.

#### Different genes – same gene terms

**Figure 5:**
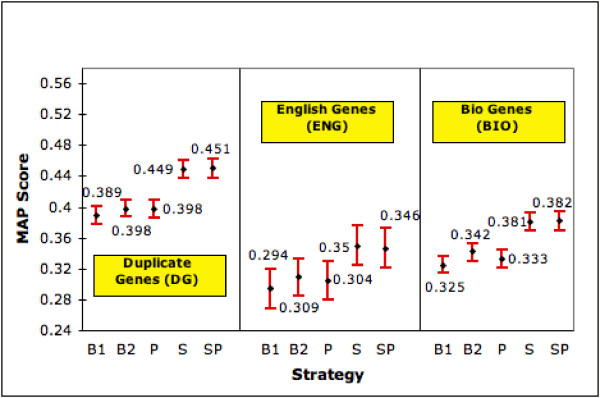
**Performance with Ambiguous Genes (MAP)**. The graph shows the MAP score (with 95% confidence interval) of each strategy on genes having duplicate records in LL (DG), general English meanings (ENG) and other biological meanings (BIO).

**Figure 6:**
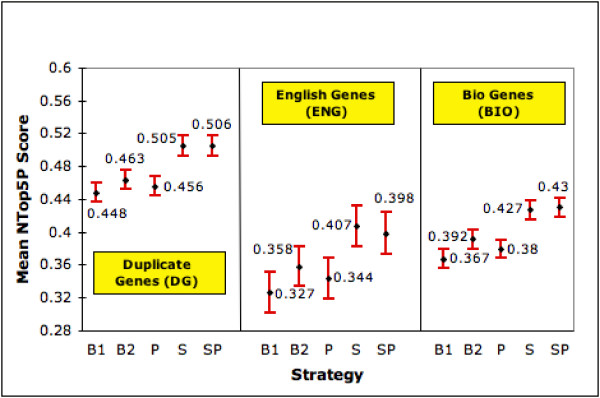
**Performance with Ambiguous Genes (NTop5P)**. The graph shows the mean NTop5P score (with 95% confidence interval) of each strategy on genes having duplicate records in LL (DG), general English meanings (ENG) and other biological meanings (BIO).

Gene terms sometimes refer to more than one gene. For example, the gene term *Frapl *refers to both a mouse gene and a rat gene. Another example is *APAH1*, which is a search term for a human gene but also is an alias for the Mouse gene *Nudt2 *.This type of ambiguity has been studied by several researches [12,13]. For each term in our 4,647 gene searches, we count the number of LL records in which it occurs. (Counts are limited to occurrences in the OFFICIAL_GENE_NAME, OFFICIAL_SYMBOL and ALIAS_SYMBOL fields and are done independent of species indicated in the record). A gene term occurring in more than 1 LL record is considered ambiguous. A gene with such a search term is considered ambiguous. Using this criteria, 2,516 of the 4,647 genes (54%) are tagged as ambiguous. Results for these 2,516 genes are presented in figures 5 &6 under 'Duplicate Genes'. We see that SP and S, still the best, improve MAP by 13.3% (15.9%) & 12.8% (15.4%) and mean NTop5P by 9.2% (12.9%) & 9.1% (12.7%) compared with B2 (B1). Since the 95% confidence intervals do not overlap, the improvements offered by both strategies are statistically significant at the 0.05 significance level.

#### Gene terms with English language meanings

The second variety of ambiguity is one where the gene term (typically gene symbols such as *GAB, ACT *and *BAR *)also has a general English language meaning. We identify such terms by a simple lookup of WordNet [27]. We eliminate instances where the meanings contain words such as gene, genome, enzyme, amino acid, which point back to a genetics related meaning. 446 of the 4,647 (9.6%) genes in our pool have at least one gene term with a general English meaning. The results for this subset are shown in figures 5 &6 under 'English Genes'. Here the S strategy takes the lead, especially in NTop5P. It gives 13.6% (19%) improvement in MAP and 13.7% (24.5%) improvement in NTop5P over B2 (B1). Except for MAP w.r.t. B2, all improvements are statistically significant at 0.05. SP also performs very well. However, the differences in MAP and mean NTop5P are in general not statistically significant at the 0.05 level.

#### Gene terms with other meanings in MEDLINE

There are also gene terms with other biological meanings [13]. For example, the gene term *ACR *has many different biomedical meanings including *albumin/creatinine ratios, acquired cellular resistance *and *acute to chronic ratio *.Meanings such as *anomalous cosmic rays *also appear in MEDLINE. The correct gene meaning we seek is *acrosin *.Other researchers have explored this aspect, especially in the context of expanding abbreviations [28,29]. In particular Schwartz and Hearst [30] published an algorithm that allows one to recognize short form-long form pairs appearing as *A *(*B*) in text where *A *is a short form and *B *is its corresponding long form. We choose this algorithm over the others [28,29] because of its simplicity and speed. It is also equivalent in effectiveness to most of the other approaches [30].

We process the retrieved documents for each gene through the Schwartz and Hearst algorithm looking for possible expansions for each term in that gene's search. 2,277/4,647 genes (47.8%) have at least 1 component gene term with more than 1 long form identified. These are considered ambiguous. This approach is not without limitations, since the algorithm relies on the presence of *A (B) *structures in the texts with *A *representing the gene term and *B *recognized correctly as a possible expansion. Results for this subset of 2,277 genes are shown under 'Bio Genes' in figures 5 &6. The SP strategy, (with S being equivalent) gives a 11.7% (17.5%) improvement in MAP and 9.7% (17.2%) improvement in mean NTop5P over B2 (B1). The figures also indicate that the improvements are significant at the 0.05 significance level. We also estimate the *extent *of ambiguity at the gene level, which we call *Ambiguity*_*Bio*_. For a gene *G *it is defined as follows:

*Ambiguity_*Bio*_*(*G*) = 0, *if *|*Expansions*(*g*_*i*_)| = 1,∀*i *∈ *n*

*Ambiguity_*Bio*_*(*G*) = ∑_*i*∈_*n*|*Expansions*(*g*_*i*_)| *otherwise*

where |*Expansion*(*g*_*i*_)| is the number of long forms for gene term *g*_*i *_found by the algorithm and *g*_*i*_*,i n *are the search terms in the PubMed query for gene G.

**Figure 7:**
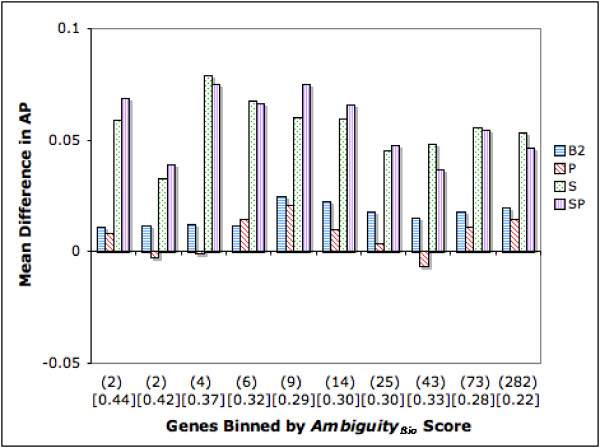
**Difference in Average Precision (AP): Genes Binned by *Ambiguity_*Bio *_*Score**. The figure depicts the relationship between *Ambiguity*_*Bio *_score (> 1) and ranking strategy performance in terms of AP. Genes are binned along the X axis by their *Ambiguity*_*Bio *_score. All bins except the right most one have 220 genes. The right most bin has 297 genes. Numbers in parenthesis below the X axis show the average *Ambiguity*_*Bio *_score for each bin. Mean B1 AP scores for each bin are shown in square brackets in the graph. The Y axis depicts the difference in performance between each strategy and B1. Bars above the X axis denote an improvement whereas bars below the X axis denote a drop in performance.

Figure 7 depicts the relationship between this ambiguity estimate (limited to genes with ambiguity score > 1) and ranking strategy performance in terms of AP. We observe improvements in the range of 9.2% to 25.8% across all the bins for the SP strategy, with >10% in 9 of the 10 bins.

#### Summary of ambiguity analysis

With two of the three varieties of ambiguity, SP is significantly better than B1 and B2 in MAP and NTop5P. The scores for ENG seem to lag behind both in MAP and NTop5P. When we consider MAP, DG seems easier to accommodate. We note that the gene sets overlap across these ambiguities. There are 411 genes that are in ENG and BIO, 361 in Eng and DG, 1,948 in DG and BIO. Thus for example, 92.2% and 80.9% of the ENG genes also fall into the BIO and DG categories respectively; 18.1% and 70.3% of the BIO genes are also in ENG and DG respectively.

### Performance versus retrieval set size

**Figure 8:**
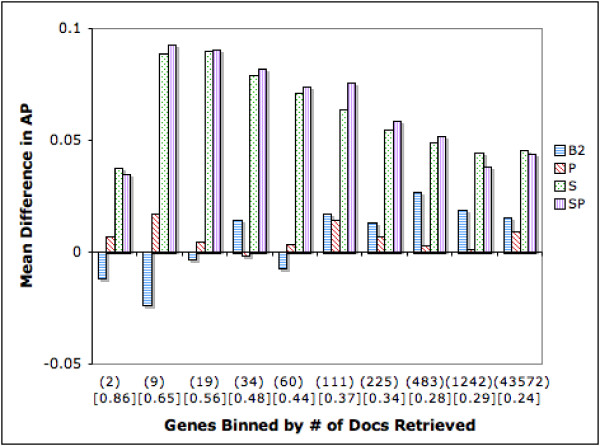
**Difference in Average Precision (AP): Genes Binned by Number of Retrieved Documents**. The figure shows the relationship between retrieved set size and ranking strategy performance in terms of AP. Genes are binned into equal sized groups based on the number of retrieved documents. Each bin, except for the last one, has 450 genes. The last bin consists of 617 genes. Average retrieved set size for each bin is shown in parenthesis and average B1 AP for each bin is shown in square brackets.

Figure 8 is a binned difference plot for AP exploring the connection with retrieved set size. We know from prior research in IR that as retrieved set size grows, precision tends to fall. We observe the same trend in terms of our baseline scores. Interestingly our S and SP strategies offer significant benefits, both against B1 and B2. Although improvements become harder to achieve as more documents are retrieved, these remain significant for all bins. The smallest improvement for SP, when 100 documents or more are retrieved, is 13.3%. The improvement in AP is 18.1% when the average retrieved set size is the largest. B2 lags significantly behind S and SP.

### Ranking results without LL summaries (Expt. 2)

**Figure 9:**
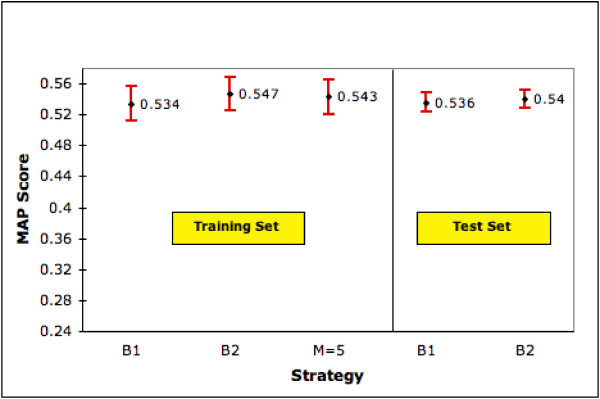
**Performance of Generic Ranking Strategy on Training Set (1,000 genes) and Test Set (3,195 genes)**. The figure shows MAP scores (with 95% confidence interval) for our generic ranking strategy, B1 and B2 on training and test sets. *M *is the number of top ranked terms selected. Since M = 5 is our best generic ranking strategy we show only the performance of this strategy.

We now focus on the 4,195 genes (of the 9,390) which do not have a summary field in LocusLink. Can we use some other strategy to rank documents for these genes? We do have the B2 ranking query (with some domain information added to the gene names). But, is there a more effective *generic *query, perhaps one that better represents the kinds of summary statements made about genes? To explore this we take the *M *most frequent words (after eliminating stopwords) from the summary fields available for the 4,647 genes (of expt. 1) and form a generic query. As with B2, the gene names are added which tailors the ranking query to each gene. Since *M *is a parameter that needs to be set, we divide our dataset of 4,195 genes randomly into a training set of 1,000 genes for training and 3,195 for testing. The training set was used to find the optimal value of *M *which was varied from 5 to 50 in steps of 5 and from 100 to 500 in steps of 50. Unfortunately none of our automatic generic strategies improve performance beyond the B2 strategy. (Thus figure 9 which shows the results for this experiment, only includes the best generic strategy with M = 5 during training). Hence we only try B1 and B2 strategies on the test set of 3,195 genes. Note that PubMed only ranks chronologically and hence comparatively our B1 and B2 performances, where ranking is by relevance potential, are themselves of value.

### Predicting B1 performance

We now return to the question left unanswered when discussing the results of experiment 1. For a given gene can we predict its B1 performance? Observe in figure 3 that as B1 performance becomes higher than approximately 0.7, our S and SP ranking strategies degrade performance. If we can identify such cases up front with reasonable accuracy, then we may avoid using the SP or S ranking strategies inappropriately. We examine four characteristics of our gene topics (DG, ENG, BIO and N: the number of documents retrieved) to see if they can be used to predict B1 performance. We begin by looking at correlations (Pearson's after data transformations) for the set of 4,647 genes (Table 1). Prior to calculating these, since the values for *Ambiguity*_*Bio *_and N are skewed, we apply a log transformation (ln(1+x)) on these values. The transformed values are referred to as N' and Bio'. Note that both ENG and DG are binary values. If the gene has at least one search term with an English meaning it gets a 1 for ENG, otherwise a 0. Likewise, a gene with at least one term shared with another gene gets a 1 for DG, otherwise a 0. N' has the strongest correlation with B1 score, followed by BIO'. Thus N' has the most potential for predicting score. Having selected N', BIO' is redundant as a feature given its strong correlation with N'. Similarly given the observed dependencies between N' on the one hand and ENG or DG on the other, we do not consider these two ambiguity properties either for prediction purposes. As an aside, one advantage with using only N' is that it is readily understood and measured (as compared with say the *Ambiguity Bio *score). Thus we run a simple least square regression model with one independent variable (N') using the equation:

*score = β*_0 _+ *β*_1_. *N'*+ ∈

where *β*_1 _is a coefficient, *β*_*o*_is a constant and ∈ the error term. Table 2 details the regression results. The coefficients are significant at extremely small *p *.Also the model is powerful with an adjusted R-square of 0.257. Thus we may be able to effectively predict the B1 AP score.

To test this calibrated model we exploit the natural split in our collection of genes. The regression model was developed on the set of 4,647 genes of experiment 1. We use the non-overlapping, naturally held out set of 4,195 genes (those without summary and product information) as our test set. We are specifically interested in predicting if the B1 score for each test gene is likely to be higher than 0.7 or not. From figure 3 observe that if B1 AP score is approximately 0.7 or higher, it is best not to do any other kind of ranking. In the test set there are 1,498/4,195 (35.7%) genes with B1 score > 0.7. The default (majority) decision that all genes will have scores ≤ 0.7, gives an accuracy of 0.643. In contrast the regression results applied to the test data gives an accuracy of 0.716, an improvement of 11.4%. These encouraging results indicate that it may be possible to predict the level of B1 performance given just the size of the retrieved set. These conclusions will be tested further in future research.

### Results with an overall strategy (Expt. 3)

**Figure 10:**
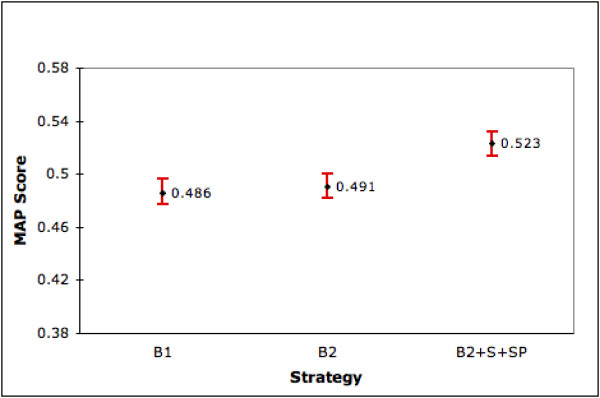
**Performance of Combined strategy over 9,390 genes (MAP)**. This figure shows the MAP scores (with 95% confidence intervals) of our combined strategy (B2+S+SP), B1 and B2 on the full set of 9,390 genes.

**Figure 11:**
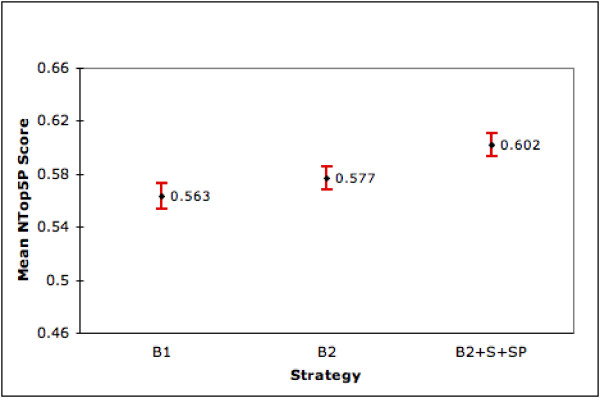
**Performance of Combined strategy over 9,390 genes (NTop5P)**. This figure shows the mean NTop5P scores (with 95% confidence intervals) of our combined strategy (B2+S+SP), B1 and B2 on the full set of 9,390 genes.

Combining the results obtained thus far, the overall strategy we propose for an arbitrary gene is to use SP to rank its retrieved set. Ranking by the S strategy is the next option. If summary is not available then we rank using the B2 strategy. In figures 10 and 11, 'B2+SP+S' shows MAP and NTop5P scores of this combined strategy on our full set of 9,390 genes (4,647 with summary and product, 4,195 without summary and 548 genes with summary alone). Compared to our two baseline strategies, we see statistically significant improvements (at the 0.05 significance level) in both performance measures. Observe that as PubMed only ranks chronologically our B1 result is itself a contribution. The ranking goal is a challenging one given that in our dataset less than 7% of the retrieved documents are known to be relevant. Additionally, we are able to improve ranking by 6.5% to 7.5% using our combined strategy even though about half of the genes in our collection do not (as yet) have summary information in LL. As this information accumulates in LL/Entrez Gene, we expect our overall performance to improve. As a refinement to our overall strategy, we may use the results from our regression model to identify genes for which ranking by B1 is preferred. This refinement will be tested in future research.

## Conclusion

We explored the relative effectiveness of five different post PubMed retrieval ranking strategies for human gene queries. We conclude that the combination of LocusLink summary and product information (or just summary) along with the gene name and aliases may be used to effectively rank retrieved documents. This conclusion is consistent with other research where some form of curated knowledge has been used to improve performance as for example the work of Koike and Takagi [16]. A ranked list of documents for each gene is provided on our web site [31].

Interestingly, using product names without summary is ineffective. This could be because the product names are more prone to being ambiguous. We find that in the absence of summary information, our manually designed generic query targeting the genetics domain combined with the gene names is the best. We were not able to automatically build a more effective 'generic' query.

Our LocusLink strategies are significantly more effective than baselines even when faced with ambiguity. The English ambiguity problem is the most challenging and is also fortunately the least prevalent. Finally, retrieved set size may give us a way to predict which genes are best handled by the B2 strategy. This could also be the basis of gene query clarity scores akin to research in [32]. We observe the presence of genes with very low B1 AP scores (< 0.2) that are not identified as having any ambiguity using our methods. Either our ambiguity detection methods are inadequate or there are other facets impeding retrieval. We plan to explore other detection approaches as seen for example in [13].

**Figure 12:**
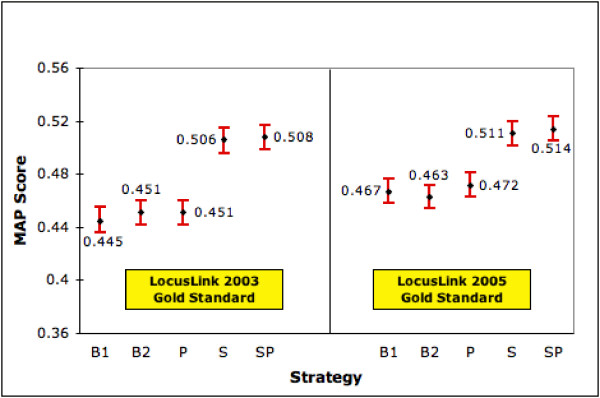
**Performance of Ranking Strategies (MAP) on Different Gold Standard Sets**. This figure shows the MAP scores (with 95% confidence interval) for each strategy on 4641 genes for which summary and product is available in two versions of LL. The left half of the graph shows the performances using relevance judgments from the 2003 LL file whereas the right half shows performances using relevance judgments from the 2005 LL file.

**Figure 13:**
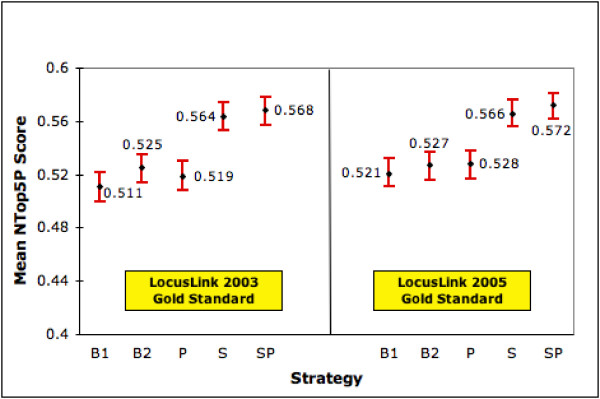
**Performance of Ranking Strategies (NTop5P) on Different Gold Standard Sets**. This figure shows the mean NTop5P scores (with 95% confidence interval) for each strategy on 4641 genes for which summary and product is available in two versions of LL. The left half of the graph shows the performances using relevance judgments from the 2003 LL file whereas the right half shows performances using relevance judgments from the 2005 LL file.

For any approach to be successful it is important for it to be robust. One criteria for robustness in our context is that S and SP continue to be relatively the best strategies even as the number of gold standard documents changes. To test this aspect we repeated our tests of the five ranking strategies using the relevance judgments from the latest LocusLink file (August 2005). We did this with the 4641 human genes that were common to the LocusLink files from 2003 (used in the experiments reported in earlier sections) and 2005 and that had both summary and product information available. The 2005 LocusLink file provides us with 45,728 relevance judgments as opposed to 29,730 in the 2003 version. Thus, we now have more than one-and-a-half times the number of relevance judgments. It is important to state that the retrieved sets for each gene were kept the same as before. That is the only difference between the two sets of experiments is that we now have one-and-a-half-times the number of relevance judgments. Figures 12 and 13 show the performances of our 5 strategies on the 4641 genes using both relevance judgment sets. We see that the relative ordering of our strategies still holds in terms of both MAP and mean NTop5P score with S and SP performing the best. As before, the improvements offered by S and SP over the baseline strategies are statistically significant at the 0.05 level. We also conducted a small expert user evaluation study. We randomly selected 34 gene topics and pooled together the top 15 ranked documents retrieved by the B2 and SP strategies for each topic. We then randomly assigned different topics to each of our 3 experts and had them judge documents for relevance. We found that the difference between SP and B2 in terms of NTop5P is 10%. This is similar to the results from other experiments.

An analysis of the probable causes of error raises some interesting observations. We observe three probable causes of failure with our SP strategy. Firstly, there are relevant documents without abstracts. SP favors documents with abstracts (as more words are then likely to be shared with the summary from LocusLink). E.g., the gene *A2M *(LLID 2) has a gold standard document (PMID 1707161) which has no abstract. This is ranked at position 5 by B2 and 180 by SP. In our dataset 25% of the gold standard instances that have abstracts are in the top 5 ranked documents (for SP) while the corresponding percentage for gold standard instances without abstracts is only 15% (also for SP). Another probable cause of error is the varying themes in the documents. Since the SP strategy is dominated by the LocusLink summary, this strategy favors documents that reflect a similar theme. For an example of an error where themes do not match, the gold standard document with PMID 9116026 for the gene *CENPB *(LLID 1059) talks about isolating a novel human homolog to the gene whereas the LL summary is primarily a description of the gene's function. This document is ranked 17th by SP and 2nd by B2. A third probable cause of error is the high rank given to some documents that mention the correct gene but in the context of another organism. E.g., for the *gastrin *gene (LLID 2520), 6 of the top 10 ranked documents (including the top ranked document) are not about humans. These are not in the gold standard set for the gene. One way of dealing with this problem may be to consider only those documents that have the MeSH term 'Human' (under the 'Organisms' semantic category), assigned to them. Although far from conclusive, these observations give us directions for more rigorous error analysis in the future.

We deliberately kept the ranking model simple using only traditional tf*idf vectors and cosine similarity. This allowed us to focus on other dimensions in this research. Clearly retrieval models such as language models with or without feedback may be tried. Having obtained encouraging results with the TREC dataset, we will now consider the full MEDLINE database. We also plan to work with genes from other genomes. We will also explore other sources for gene descriptions such as OMIM. These may offer interesting avenues for genes without LL/Entrez Gene summaries.

## Methods

### Gene queries and documents

We start with 12,385 human genes with known function identified from LocusLink (LL)^1^. LL is a manually curated database with a variety of information on genes such as names, symbols and pointers to relevant documents. For each gene we search MEDLINE using the ESearch utility available from the NCBI website [33]. Our search strategy is the disjunction of the aliases for the gene, taken from the OFFICIAL_GENE_NAME, OFFICIAL_SYMBOL and ALIAS_SYMBOL fields of LL. For example the search for the gene with official name *alpha- 1-B glycoprotein *is "A1BG OR A1B OR ABG OR GAB OR alpha-1-B glycoprotein". Relevant documents (our gold standard) are identified by extracting the documents identified in each gene's LL record (PMID and GRIF fields). Documents listed in these fields are typically identified by human curators and indexers (with subject expertise).

In order to minimize the load on the NCBI server, given our large number of queries, we constrain our experiments to the 2004 3TREC Genomics dataset [24], which contains close to 4.6 million MEDLINE records. This subset is a recent one-third (approximately 1994 to 2003) of the full MEDLINE database. When limited to this dataset, 9,390 of the 12,385 original gene topics have at least 1 retrieved relevant document. These retrieve a total of 45,216,725 records from this dataset (average = 4,815). The one modification made to the TREC dataset was to add relevant documents retrieved but not already present (5,516 relevant documents). 4,111,272 unique records (of the 4.6 million) in the modified TREC dataset were retrieved for at least 1 gene query. The 9,390 genes and their associated data form the basis of our experiments.

Table 3 gives the distribution of the retrieved and relevant document set sizes. Observe that 44% of the topics retrieve 100 or more documents while almost 25% retrieve 500 or more documents. At the same time 76% of the topics have five or less relevant documents.

### Ranking system

We use Lemur version 3.1 [34] installed on a system with 2 GB RAM, running Redhat Linux 9.0. Lemur is a toolkit developed by researchers at Carnegie Mellon University and the University of Massachusetts for language modeling and IR-related tasks. For a given gene its retrieved documents and its ranking query(ies) are first represented by term vectors where term weighting is done using a basic tf*idf (term frequency * inverse document frequency) strategy. These vectors are built using Lemur. A stoplist of 571 commonly used English words (such as 'a', 'are', 'the') is used and words are stemmed. Cosine similarity scores in [0,1] are calculated between query vectors and document vectors. Given a ranking query and a retrieved set of documents for a gene, the documents are ranked by their cosine similarity score with the query vector. The ranked sets are limited to the top ranked 10,000 documents. We believe it is unlikely that a user would want a larger retrieved set. To index the documents we use the title, abstract, MeSH (Medical Subject Headings) and RN (chemical names) fields.

## Availability

A web-based system offering access to retrieved and ranked document sets for gene queries is freely available at ().

## Authors' contributions

AKS and PS were both equally involved in designing the experiments, analyzing the results and writing the paper. Additionally, AKS was responsible for running all the experiments.

## Note

^1 ^Download date October 2, 2003. We note that LocusLink is now a part of NCBI's Entrez Gene. Importantly, the LocusLink fields used in this study are still available through Entrez Gene.

## Figures and Tables

**Table 1:** Correlation Coefficients. The table shows the strength of the correlations among the different kinds of ambiguities and the number of retrieved documents and their correlation with the B1 AP Score.

	**DG**	**Bio'**	**Eng**	**N'**
**Bio'**	0.371			
**Eng**	0.175	0.456		
**N'**	0.336	0.697	0.436	
**B1 AP Score**	-0.182	-0.363	-0.149	-0.508

**Table 2:** Regression Results. The results of the regression to predict the B1 AP score using the size of the retrieved set as the predictive variable.

**Model**	*β*	**std err**	**t**	**P**
Constant (*β*_0_)	0.783	0.009	83.23	0
N(*β*_1_)	-0.071	0.002	-40.14	9.314E-303

R-square: 0.258, Adjusted R-square: 0.257

**Table 3:** Distribution of Retrieved and Relevant Documents (9,390 genes) A topic is defined as a gene query. Thus, in the table, 5270 gene queries retrieve between 0–100 documents and 7101 gene queries have between 1–5 relevant documents identified in LocusLink.

**# Retrieved Documents**	**# Topics**	**# Relevant Documents**	**# Topics**
0–100	5270 (56%)	1–5	7101 (76%)
101–500	1944 (21%)	6–10	1344 (14%)
500–1000	633 (7%)	11–15	430 (5%)
1001–2500	676 (7%)	16–20	204 (2%)
2501–5000	323 (3%)	21–25	100 (1%)
5001–10000	230 (2%)	26–30	58 (<1%)
10001–25000	154 (2%)	31–35	38 (<1%)
25001–50000	71 (1%)	36–40	29 (<1%)
> 50,000	89 (<1%)	> 40	86 (<1%)

## References

[B1] Adamic LA, Wilkinson D, Huberman BA, Adar E (2002). A literature based method for identifying gene-disease connections. Proceedings of the 1st IEEE Computer Society Bioinformatics Conference.

[B2] Rindflesch TC, Tanabe L, Weinstein JN, Hunter L (2000). EDGAR: Extraction of drugs, genes, and relations from biomedical literature. Proceedings of the Pacific Symposium on Biocomputing (PSB).

[B3] Shatkay H, Edwards S, Wilbur WJ, Boguski M (2000). Genes, Themes, and Microarrays: Using Information Retrieval for Large-Scale Gene Analysis. Proceedings of the Eighth International Conference on Intelligent Systems for Molecular Biology (ISMB).

[B4] Raychaudhuri S, Altman RB (2003). A literature-based method for assessing the functional coherence of a gene group. Bioinformatics.

[B5] Kankar P, Adak S, Sarkar A, Murari K, Sharma G (2002). MedMesh Summarizer: Text Mining for Gene Clusters. Proceedings of the 2nd SIAM International Conference on Data Mining.

[B6] Wren JD, Garner HR (2004). Shared relationship analysis: ranking set cohesion and commonalities within a literature-derived relationship network. Bioinformatics.

[B7] Chaussabel D, Sher A (2002). Mining microarray expression data by literature profiling. Genome Biol.

[B8] Hirschman L, Morgan AA, Yeh AS (2002). Rutabaga by any other name: extracting biological names. J Biomed Inform.

[B9] Tanabe LK, Wilbur WJ (2002). Tagging gene and protein names in full text articles. Proceedings of the Workshop on Natural Language Processing in the Biomedical Domain.

[B10] Morgan A, Hirschman L, Yeh A, Colosimo M (2003). Gene Name Extraction Using FlyBase Resources. Proceedings of the ACL 2003 Workshop on Natural Language Processing in Biomedicine.

[B11] Weeber M, Schijvenaars BJA, van Mulligen EM, Mons B, Jelier R, van der Eijk C, Kors JA (2003). Ambiguity of Human Gene Symbols in LocusLink and MEDLINE: Creating an Inventory and a Disambiguation Test Collection. Proceedings of the AMIA Symposium.

[B12] Tuason O, Chen L, Liu H, Blake JA, Friedman C (2004). Biological Nomenclatures: A Source of Lexical Knowledge and Ambiguity. Proceedings of the Pacific Symposium on Biocomputing (PSB).

[B13] Chen L, Liu H, Friedman C (2005). Gene Name Ambiguity of Eukaryotic Nomenclatures. Bioinformatics.

[B14] Liu H, Lussier YA, Friedman C (2001). Disambiguating ambiguous biomedical terms in bio medical narrative text: an unsupervised method. Journal of Biomedical Informatics.

[B15] Podowski RM, Cleary JG, Goncharoff NT, Amoutzias G, Hayes WS (2005). Suregene, a scalable system for automated term disambiguation of gene and protein names. Journal of Bioinformatics and Computational Biology.

[B16] Koike A, Takagi T (2004). Gene/Protein/Family Name Recognition in Biomedical Literature. Proceedings of the HLT-NAACL 2004 Workshop: BioLINK Linking Biological Literature, Ontologies and Databases.

[B17] Seki K, Mostafa J (2003). A Probabilistic Model for Identifying Protein Names and their Name Boundaries. Proceedings of the 2nd IEEE Computer Society Bioinformatics Conference.

[B18] Schijvenaars B1, Mons B, Weeber M, Schuemie MJ, van Mulligen EM, Wain HM, Kors JA (2005). Thesaurus-based disambiguation of gene symbols. BMC Bioinformatics.

[B19] KDD Cup 2002. http://www.biostat.wisc.edu/~craven/kddcup/.

[B20] Hirschman L, Yeh A, Blaschke C, A V (2005). Overview of BioCreAtIvE: critical assessment of information extraction for biology. BMC Bioinformatics.

[B21] TREC Genomics Track. http://ir.ohsu.edu/genomics/.

[B22] Blaschke C, Leon EA, Krallinger M, Valencia A (2005). Evaluation of BioCreAtIvE assessment of task 2. BMC Bioinformatics.

[B23] Hersh W, Bhupatiraju RT (2003). TREC Genomics Track Overview. Proceedings of The 12th Text Retrieval Conference (TREC).

[B24] Hersh W, Bhupatiraju RT, Ross L, Johnson P, Cohen AM, Kraemer DF (2004). TREC 2004 Genomics Track Overview. Proceedings of The 13th Text Retrieval Conference (TREC).

[B25] Singhal A, Mitra M, Buckley C (1997). Learning routing queries in a query zone. Proceedings of the 20th ACM SIGIR Conference.

[B26] Maglott D (2003). LocusLink: A Directory of Genes. The NCBI Handbook, NCBI.

[B27] WordNet – Princeton University Cognitive Science Laboratory. http://wordnet.princeton.edu.

[B28] Chang JT, Schütze H, Altman RB (2002). Creating an Online Dictionary of Abbreviations from MEDLINE. J Am Med Inform Assoc.

[B29] Pustejovsky J, Castano J, Cochran B, Kotechi M, Morrell M (2001). Automatic extraction of acronym-meaning pairs from MEDLINE databases. Proceedings of Medinfo.

[B30] Schwartz AS, Hearst MA (2003). A Simple Algorithm for Identifying Abbreviation Definitions in Biomedical Text. Proceedings of the Pacific Symposium on Biocomputing (PSB).

[B31] Retrieval for Gene Queries. http://sulu.info-science.uiowa.edu/genedocs/.

[B32] Cronen-Townsend S, Zhou Y, Croft WB (2002). Predicting query performance. Proceedings of the 25th ACM SIGIR Conference.

[B33] ELink Entrez Utility. http://eutils.ncbi.nlm.nih.gov/entrez/query/static/elink_help.html.

[B34] Lemur Project. http://www-2.cs.cmu.edu/~lemur/.

